# The complete chloroplast genome of *Rubus rosifolius* (Rosaceae), an ornamental and medicinal plant

**DOI:** 10.1080/23802359.2022.2093669

**Published:** 2022-07-18

**Authors:** Xuelian Yang, Xia Wang, Yongfei Wu, Li Yan, Xiaojing Hu, Wanping Zhang

**Affiliations:** College of Agriculture, Guizhou University, Guiyang, Guizhou Province, China

**Keywords:** *Rubus rosifolius*, chloroplast genome, Rosaceae, Illumina sequencing

## Abstract

*Rubus rosifolius* belongs to the genus *Rubus* in the family Rosaceae and is widely distributed globally. It has white flowers and red fruits. Moreover, it has medicinal value for diseases of the stomach and other areas. However, the complete chloroplast (cp) genome of *R. rosifolius* remains unclear. In the present study, we sequenced the complete cp genome of *R. rosifolius* (GenBank accession no. OL435124), which had a typical quadripartite structure and a size of 155,650 bp. Fifteen genes (*trn*K-UUU, *rps*16, *trn*G-UCC, *atp*F, *rpo*C1, *trn*L-UAA, *trn*V-UAC, *pet*B, *pet*D, *rpl*16, *rpl*2, *ndh*B, *trn*I-GAU, *trn*A-UGC, and *ndh*A) contained an intron, and two genes (*clp*P and *ycf*3) contained two introns. The gene *rps*12 showed trans-splicing. Phylogenetic analysis showed that *R. rosifolius* was closely related to *Rubus taiwanicola, Rubus rubroangustifolius*, and *Rubus glandulosopunctatus.*

*Rubus rosifolius* Sm. 2010 belongs to the genus *Rubus* in the family Rosaceae and is widely distributed globally. This species is native to the Solomon Islands, New Caledonia, Vanuatu (eastern coastal Australia), and Mauritius (southern Indonesia), Indochina, and China (Quadros et al. 2020). *R. Rosifolius* produces beautiful ornamental white flowers and red fruit. *R. rosifolius* is used in traditional medicine to treat diarrhea and stomach diseases and has analgesic, antimicrobial, antihypertensive, and other pharmacological properties (Quadros et al. 2020). Thus, genetic and genomic information on *R. rosifolius* is vital for systematic research and conservation. However, the complete chloroplast (cp) genome of *Rubus rosifolius* remains unclear. In this study, we sequenced the complete cp genome of *R. rosifolius* to promote further study and conservation.

Samples of *R. rosifolius* were collected from the Guizhou Botanical Garden, Guiyang, Guizhou Province, China (26°37'20″N, 106°43'29″E). The College of Agriculture approved this study, which was performed under the National Wild Plant Protective Regulations. The voucher specimen (KXP20210701YX) was deposited at the Laboratory of the College of Agriculture at Guizhou University, Guiyang (contact person: Xuelian Yang, email: yxl1299927812@outlook.com). Total genomic DNA was extracted from 300 mg of fresh leaves using the CTAB method. Libraries with an average length of 350 bp were constructed using a NexteraXT DNA library preparation kit. The Illumina Novaseq 6000 platform was used to sequence these libraries and create raw sequence data. After editing using the NGS QC Tool Kit v2.3.3 (Patel and Jain 2012), we assembled the complete cp genome from 3.82 G of high-quality data using the *de novo* assembler SPAdes v3.11.0 (Bankevich et al. 2012). Finally, the complete cp genome was annotated using the PGA software (Qu et al. 2019).

The complete cp genome of *R. rosifolius* (GenBank accession no. OL435124) had a typical quadripartite structure and a size of 155,650 bp, including two inverted repeats (IRs; 25,748 bp each), a large single-copy region (85,443 bp), and a small single-copy region (18,711 bp). In this study, 131 genes in the cp genome were annotated, including 86 protein-coding genes (PCGs), 37 tRNA genes, and eight rRNA genes. Among them, we found that five PCGs (*rpl*2, *rpl*23, *ycf*2, *ndh*B, and *rps*7), four rRNA genes (*rrn*16, *rrn*23, *rrn*4.5, and *rrn*5), and seven tRNA genes (*trn*L-CAU, *trn*L-CAA, *trn*V-GAC, *trn*I-GAU, *trn*A-UGC, *trn*R-ACG and *trn*N-GUU) were duplicated in the IR regions. The overall GC content was 36.9%. The total sizes of the PCGs and RNA genes (tRNAs and rRNAs) were 80,334 and 11,839 bp, respectively; their GC contents were 37.71 and 54.91%, respectively. Thus, the GC content of the RNA genes was much higher than that of the PCGs and the complete cp genome. Furthermore, 15 genes (*trn*K-UUU, *rps*16, *trn*G-UCC, *atp*F, *rpo*C1, *trn*L-UAA, *trn*V-UAC, *pet*B, *pet*D, *rpl*16, *rpl*2, *ndh*B, *trn*I-GAU, *trn*A-UGC, and *ndh*A) contained an intron, and two genes (*clp*P and *ycf*3) contained two introns. Additionally, the gene *rps*12 had trans-splicing.

To analyze the phylogenetic relationships of *R. rosifolius* within the genus *Rubus*, we aligned the 74 homologous protein-coding genes in each of the 34 complete cp genomes from NCBI using the MAFFT 7.037 software (Katoh and Standley 2013) and the FFT-NS-2 strategy. Subsequently, we used the model-finder var 1.6 to select the TVM + F + I + G4 model (Kalyaanamoorthy et al. 2017). The phylogenetic tree was constructed using the RAxML var 8.2.9 software (Stamatakis 2014) and 1000 bootstrap replicates, based on the maximum likelihood method. The results showed that *R. rosifolius* is closely related to *Rubus taiwanicola* (NC_057631.1), *Rubus glandulosopunctatus* (NC_057624.1), and *Rubus rubroangustifolius* (NC_057629.1), with high bootstrap values (>90) (Figure 1).

**Figure 1.**
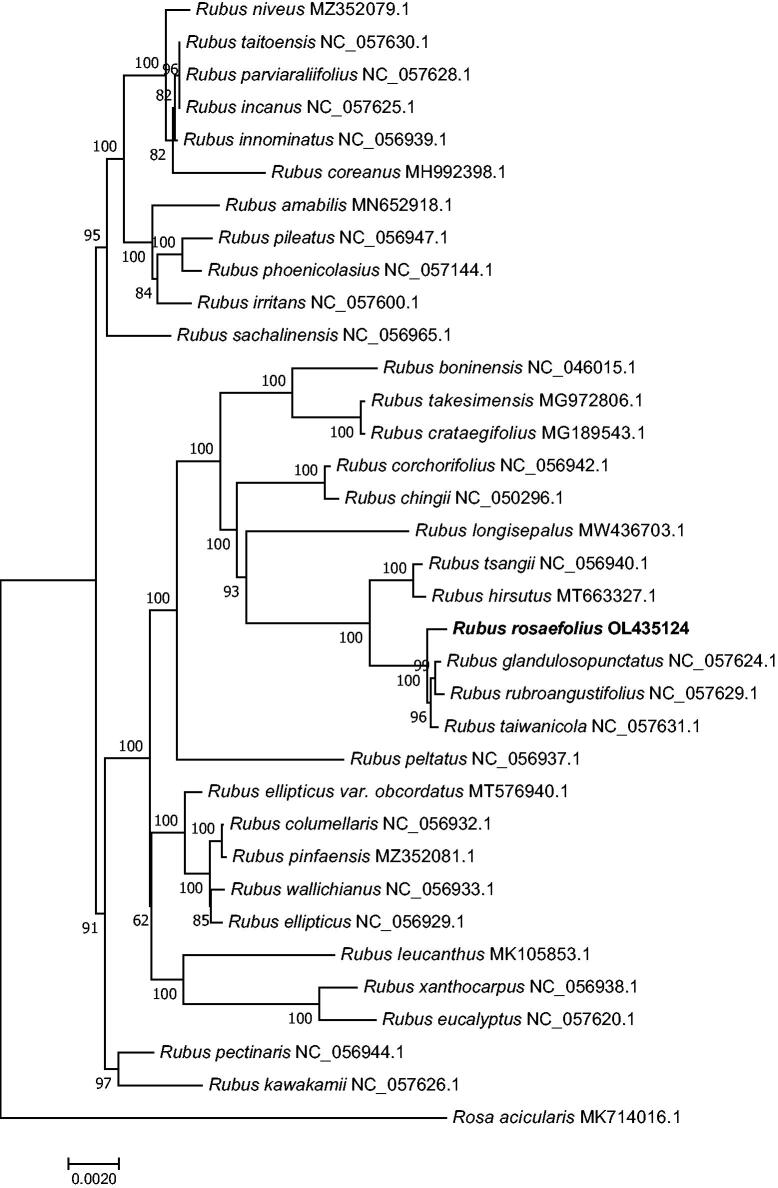
The maximum likelihood phylogenetic tree is based on 74 homologous protein-coding genes in 34 species. *Note*: Numbers to the right of the nodes represent the bootstrap value for 1000 replicates.

## Data Availability

The genome sequence data supporting the findings of this study are available in the NCBI GenBank (https://www.ncbi.nlm.nih.gov/) under accession no. OL435124. The associated BioProject, SRA, and Bio-Sample numbers were PRJNA786327, SRR17183907, and SAMN23667426, respectively.
